# 3D Bioprinting and Artificial Intelligence for Tumor Microenvironment Modeling: A Scoping Review of Models, Methods, and Integration Pathways

**DOI:** 10.1021/acs.molpharmaceut.5c01062

**Published:** 2025-09-18

**Authors:** Urszula Piotrowska, James Tsoi, Pradeep Singh, Avijit Banerjee, Marcin Sobczak

**Affiliations:** † Department of Pharmaceutical Chemistry and Biomaterials, Medical University of Warsaw, 1 Banacha Str., 02-097 Warsaw, Poland; ‡ Division of Applied Oral Sciences and Community Dental Care, Faculty of Dentistry, The University of Hong Kong, Hong Kong SAR 999077, China; § Centre of Oral Clinical Translational Sciences, Faculty of Dentistry, Oral & Craniofacial Sciences, King’s College London, London WC2R 2LS, United Kingdom

**Keywords:** 3D bioprinting, tumor microenvironment, machine learning, deep learning, artificial intelligence, colorectal cancer, oral cancer, breast cancer, glioma cancer

## Abstract

Recent advances in cancer research emphasize the development of physiologically relevant models to better understand tumor behavior and therapeutic responses. The tumor microenvironment (TME) plays a pivotal role in tumor progression, metastasis, and treatment resistance. Three-dimensional (3D) bioprinting offers unique capabilities for constructing complex in vitro tumor models that closely replicate the TME heterogeneity and interactions. These biomimetic models surpass the limitations of traditional 2D cultures and reduce the reliance on animal testing. This review aimed to systematically map current research on 3D bioprinting and artificial intelligence (AI) applications in modeling TME across selected cancer types. The review was structured into three thematic domains: 3D bioprinting of TME models for selected cancer types, AI applications in 3D bioprinting regardless of clinical focus, and integration of AI with 3D bioprinting specifically for TME modeling. A comprehensive literature search was conducted in PubMed, covering publications from January 2020 to June 2025. The review was conducted in accordance with PRISMA-ScR guidelines and focused on peer-reviewed original research articles published in English. Included cancer types were colorectal cancer, oral cancer, breast cancer, and glioma. In total, 63 articles were screened for TME-specific 3D bioprinting, with 44 included. For AI applications in 3D bioprinting irrespective of cancer type, 67 records were identified and 14 met the inclusion criteria. Only one study explicitly integrated AI and 3D bioprinting for TME modeling, highlighting a critical research gap. These findings are illustrated in the PRISMA flowcharts for clarity. Despite growing interest in both 3D bioprinting and AI, their combined application for modeling of the tumor microenvironment remains limited. The reviewed literature demonstrates significant progress in bioink development, process optimization, and quality control through AI methods. However, further interdisciplinary research is necessary to realize the potential of AI in enhancing TME modeling for oncology applications.

## Graphical abstract

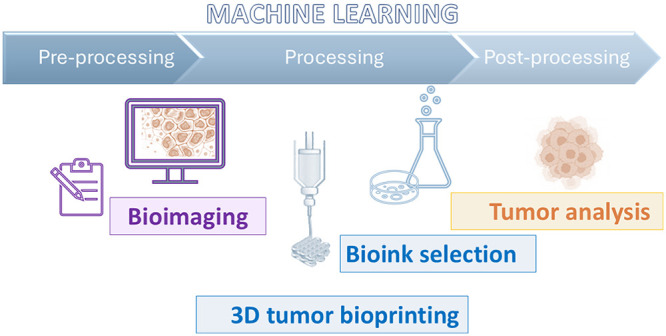

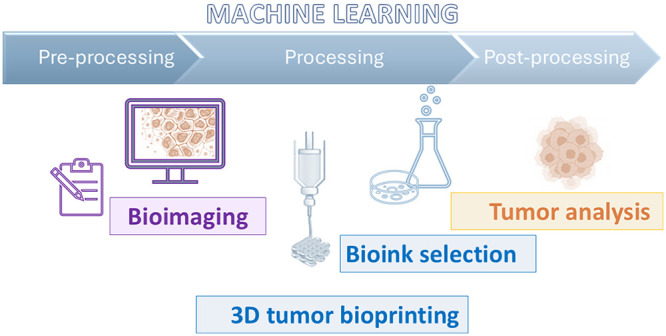

## 1 Introduction

Cancer remains a major public health issue worldwide. The *GLOBOCAN 2022* report by the International Agency for Research on Cancer (IARC) estimates that there were approximately 20 million new cancer cases and 9.7 million cancer deaths globally in 2022.^1^ This updated data captures trends influenced by the COVID-19 pandemic, whose effects on cancer incidence, screening, and treatment access are still being examined. In the US, the American Cancer Society projects that in 2024 there will be 2,001,140 new cancer cases and 611,720 cancer deaths.^2^

Models that closely resemble in vivo tumor environments are essential for understanding tumor biology and developing effective therapies. Traditional approaches, such as 2D cell cultures and animal-based systems, have long served as foundational tools in cancer research. However, these models often fail to replicate the complex spatial, biochemical, and cellular interactions that characterize the human tumor microenvironment (TME). While xenograft and genetically engineered mouse models offer certain advantages, they still fall short in mimicking the dynamic and heterogeneous conditions found in human tumors.^3–5^ Furthermore, for many aggressive and highly lethal cancer types, suitable in vivo representations of cancers remain lacking.

Recent advances in tissue engineering, particularly three-dimensional (3D) bioprinting, have significantly improved the development of physiologically relevant tumor models,^6^ e.g., lung,^7,8^ breast,^9,10^ colorectal,^11^ liver,^12^ glioma,^13^ or bladder cancer.^14^ As such, 3D bioprinting has become a valuable tool in preclinical drug testing and the development of personalized therapeutic approaches.^15,16^ However, replicating the TME’s complex cellular interactions and signaling dynamics remains a key challenge.

Artificial intelligence (AI), particularly through machine learning (ML) and deep learning (DL), offers promising solutions by enhancing the design and predictive capabilities of 3D bioprinted models. AI enhances printing precision, expands the diversity of printable materials, and improves postprinting cell viability. ML algorithms can analyze high-dimensional data generated from bioprinted constructs to predict drug responses, refine printing parameters, and optimize design strategies.^17^ Additionally, DL techniques integrated with imaging technologies such as CT and MRI support advanced tumor characterization and model validation.

In light of the rapid technological convergence between AI and 3D bioprinting, a scoping review was conducted. The objective was to systematically map the current research landscape concerning the use of 3D bioprinting and AI in modeling the TME. The review focuses on four biologically complex and clinically significant cancer types: colorectal (CRC), oral, breast (BrCa), and glioma (including glioblastoma, GBM), which are characterized by highly heterogeneous TMEs. These cancers were selected based on their high global prevalence and clinical relevance, as well as the heterogeneity of their TME. CRC and BrCa represent two of the most common malignancies worldwide, with extensive efforts to develop physiologically relevant in vitro models. Oral cancers are characterized by a complex TME influenced by stromal and immune interactions, while gliomas, particularly GMB, remain among the most aggressive and therapy-resistant tumors in which TME-driven heterogeneity plays a critical role. These tumor types therefore provide representative examples of diverse TME characteristics while at the same time reflecting areas where in vitro bioprinted models are being actively developed.

## 2 Methods

This scoping review was conducted in accordance with the PRISMA-ScR (Preferred Reporting Items for Systematic Reviews and Meta-Analyses extension for Scoping Reviews) guidelines.^18^ The objective was to systematically map the current research landscape on the use of 3D bioprinting and artificial AI in modeling the TME. The review was structured around three thematic domains:

1. 3D bioprinting of TME models for selected cancer types (Section 4).
2. AI applications in 3D bioprinting, regardless of clinical focus (Section 5).
3. Integration of AI and 3D bioprinting with focus on TME modeling (Section 6).

This structure enabled a focused exploration of individual domains and their intersections, helping to identify key trends, synergies, and research gaps.

### 2.1 Scope

The scope of this review was limited to four biologically and clinically significant cancer types: CRC, oral cancer, BrCa, and glioma. These were selected based on the availability of experimental models, the importance of their TME in disease progression, and their prominence in the bioprinting literature.

Research questions were developed for each thematic section:

- What strategies and biofabrication methods are used to model the TME in these cancers via 3D bioprinting?
- How is AI applied in the context of 3D bioprinting technology?
- Are there existing studies that integrate AI-driven approaches into the 3D bioprinting of TME models, and if so, for which cancer types?

### 2.2 Search Strategy

A comprehensive literature search was conducted in the PubMed database, covering the period from January 1, 2020 to June 30, 2025. The search strategy combined keywords and MeSH terms related to *3D bioprinting biofabrication*, *bioink*, *tumor microenvironment*, *artificial intelligence*, *machine learning*, *deep learning*, and *process control*, as well as cancer-specific terms (*colorectal cancer*, *oral cancer*, *breast cancer*, *glioma*, and *glioblastoma*).

The detailed Boolean search expressions used for each thematic section of the review are provided in the Supporting Information (Table S1). Only articles published in English were considered. Additionally, the reference lists of the included articles were manually screened to ensure completeness.

### 2.3 Criteria for Selection and Evaluation

The eligibility criteria were defined according to the PCC framework, as recommended for scoping reviews. The *Population* of interest included in vitro models of colorectal, oral, breast, and glioma/glioblastoma cancers. The *Concept* referred to the use of 3D bioprinting techniques and, where applicable, the integration of AI methods, including ML and DL, in the context of TME modeling. The *Context* comprises biomedical and bioengineering studies focused on reconstructing the TME for experimental, diagnostic, or therapeutic purposes.

To be included, studies were required to be original, peer-reviewed research articles or preprints published in English between January 1, 2020, and June 30, 2025. Eligible studies had to employ 3D bioprinting techniques relevant to the selected TMEs and, when applicable, incorporate AI components to assist in design, fabrication, or analysis.

Exclusion criteria were applied to nonoriginal publications (e.g., reviews, editorials, and letters), studies published outside the defined period, articles not in English, and studies that did not address 3D bioprinting, TME modeling, or AI applications in the context relevant to this review.

After the final selection, each study was categorized according to its primary focus—bioprinting alone, AI alone, or their integration, allowing structured analysis across cancer types and identification of both isolated and combined applications of these technologies.

### 2.4 Data Screening and Extraction

The study selection and data extraction process followed the PRISMA 2020 guidelines. Two independent reviewers screened all of the retrieved records for eligibility. At the identification stage, records were excluded upfront if they violated predefined inclusion criteria, specifically when they were published outside the study period, not in English, or classified as nonoriginal research (e.g., reviews, editorials, letters). For TME-related searches, several records (n = 16) were removed at this stage based on metadata (year, language, and publication type). In contrast, for AI-related searches, no records were excluded during identification, as the relevance and study type could only be determined after abstract or full-text review.

The remaining records were assessed in two phases. During title and abstract screening, clearly irrelevant studies were removed. Subsequently, a full-text assessment was performed for all articles not excluded at the screening stage. At this step, studies were excluded if they did not address 3D bioprinting, TME modeling, or AI applications in the context relevant to this review.

The eligibility process resulted in a final set of included articles, which were then subjected to systematic data extraction. For each study, the following information was charted: authors, year of publication, country, study design, cancer type, bioprinting method, biomaterials or bioinks used, and, where applicable, AI methods and their applications (e.g., predictive modeling, optimization of bioinks, or process control).

All extracted data were synthesized into structured summary tables and illustrative figures, enabling a comparative analysis of research trends across different cancer types, bioprinting strategies, and AI-assisted approaches.

The full selection process, including the number of records identified, excluded at each stage, and studies included in the review, is documented in the PRISMA 2020 flowcharts (Figure 1 and 2).

**Figure 1:**
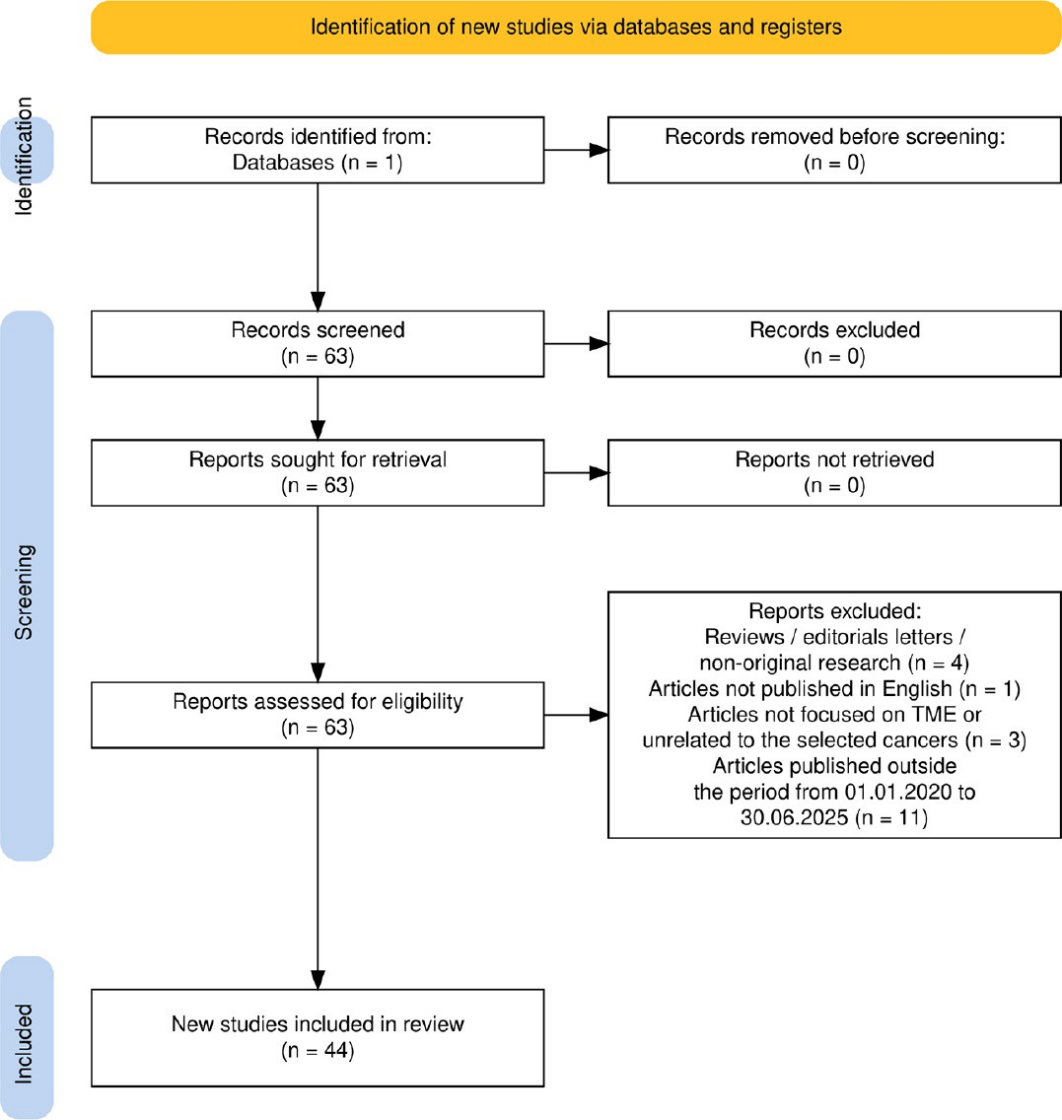
PRISMA 2020 flow diagram illustrating the study selection process for *3D bioprinting of TME models for selected cancer types*. The diagram was generated using the PRISMA2020 R package and Shiny app.^19^

**Figure 2:**
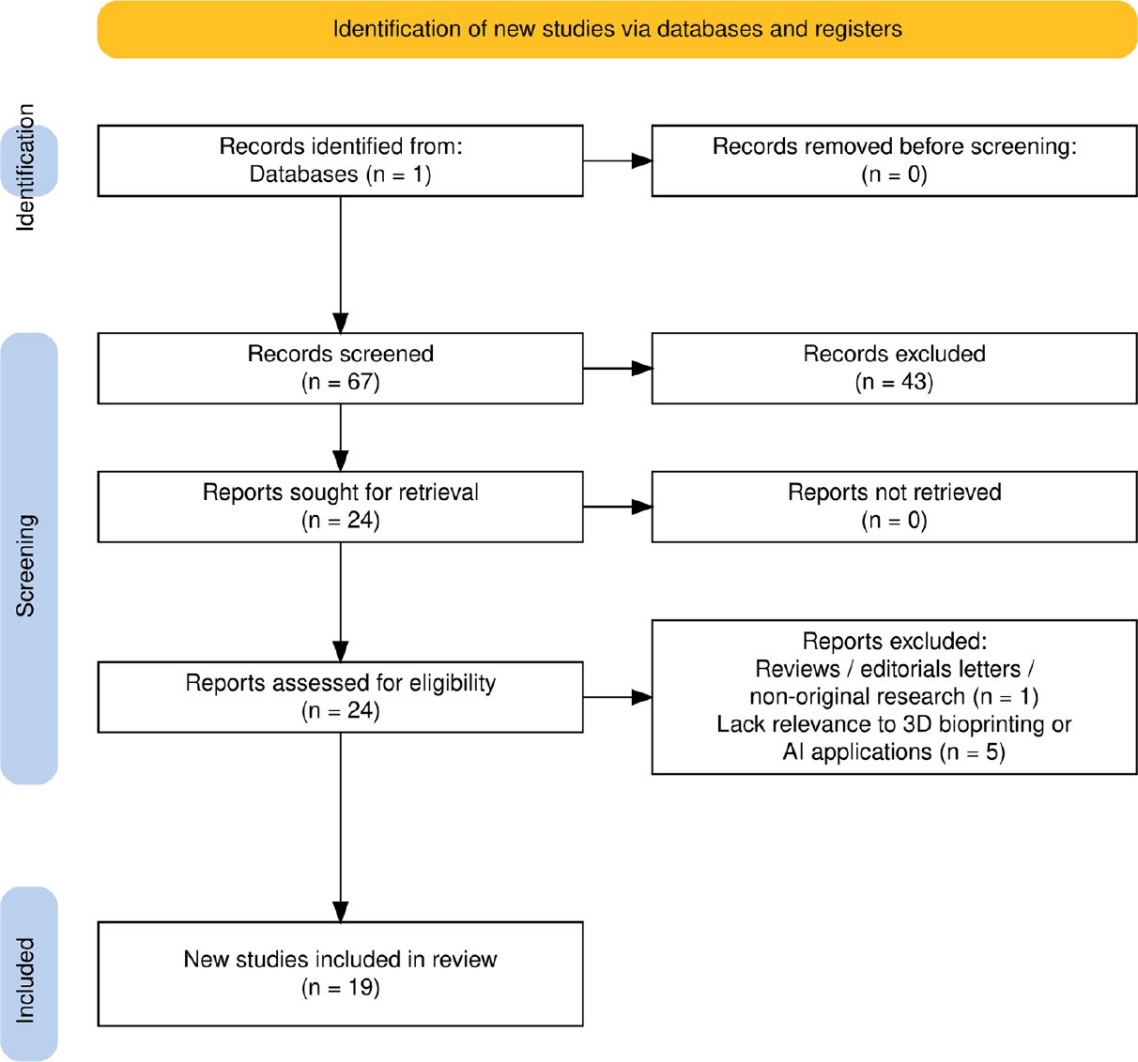
PRISMA 2020 flow diagram illustrating the study selection process for *AI applications in 3D bioprinting*, *regardless of clinical focus*. The diagram was generated using the PRISMA2020 R package and Shiny app.^19^

## 3 Key Concepts and Definitions

Given the interdisciplinary nature of this article, it is essential to clarify key terms that recur throughout the analysis. Defining concepts such as 3D bioprinting, bioink, biomaterial ink, artificial intelligence, tumor microenvironment, and personalized medicine provides the necessary context for understanding their roles in cancer modeling and therapeutic development. The definitions included in Table 1 are based on current, peer-reviewed scientific literature and authoritative sources.

**Table 1:** Definitions of Key Terms Used in the Article

**Key term**	**Definition**	**Ref.**
**3D bioprinting**	A biofabrication technology that uses layer-by-layer deposition of bioinks composed of living cells, biomaterials, and biologically active factors to construct tissue-like structures with defined spatial architecture.	^20,21^
**Bioink**	A formulation of cells combined with biomaterials and cell-containing hydrogels that mimics the extracellular matrix and possesses printability, biocompatibility, and structural integrity necessary for use in bioprinting.	^21–23^
**Biomaterial ink**	A formulation that include biologically active components but no cells, which can be seeded after printing.	^24^
**Artificial intelligence**	A field of computational science focused on developing systems capable of performing tasks that normally require human intelligence, such as learning, pattern recognition, and decision-making.	^25^
**Machine learning**	A subfield of AI involving algorithms that learn patterns from data and improve performance over time without being explicitly programmed.	^26^
**Deep learning**	An advanced form of ML using artificial neural networks with multiple layers to model complex data structures, especially useful in image analysis and predictive modeling.	^27^
**Tumor microenvironment**	A complex system of cancer cells, stromal cells, immune cells, vasculature, extracellular matrix, and signaling molecules that together influence tumor progression and therapy resistance.	^28^
**Personalized medicine**	An individualized approach to medical treatment based on genetic, molecular, and phenotypic information, aiming to optimize efficacy and reduce adverse effects.	^29^

### 3.1 3D Bioprinting and Cell Sheet Engineering

3D bioprinting typically utilizes hydrogels or solid scaffolds to better mimic the structural and physiological properties of living tissues. In this approach, cells are incorporated into temporary, removable biomaterial scaffolds and printed into three-dimensional constructs.^30^ An alternative method leverages the natural ability of cells to self-organize into 3D structures without the need for exogenous materials. This technique can generate tissue layers where cells naturally interact and self-organize, replicating the hypoxic microenvironment typically found at the core of tumor constructs due to limited oxygen diffusion. Such conditions are valuable for studying hypoxia-induced processes, including tumor progression and drug resistance.^31,32^

### 3.2 Bioinks and Biomaterials

The definition of a bioink presented in Table 1 varies, encompassing a combination of cells and biomaterials, mixtures of materials and cells, printable materials used during cell printing, cell-containing hydrogels, materials that offer both printability and cytocompatibility, and those that simulate an extracellular matrix (ECM) environment.^33^ The ECM provides structural support for tumor cells and regulates cell–cell and cell–matrix interactions, acting as a critical cue to direct tumorigenesis and metastasis^34^ or modulating the response to therapy.^35^ Spheroids and strands, forms of cell aggregates, are also commonly used as bioinks in the fabrication of 3D-printed tissue constructs.^36^ During the printing process, these cellular aggregates are deposited as cylindrical or spherical units, each with a diameter between 260 and 500 μm.^37^

### 3.3 Machine Learning and Deep Learning

ML enables computers to learn and analyze data autonomously. The process begins by inputting large data sets into algorithms, which assist in making predictions or decisions. ML is broadly classified into three types: *supervised learning*, where models are trained using labeled data with known input-output relationships; *unsupervised learning*, which uncovers patterns in unlabeled data; and *reinforcement learning*, which employs trial-and-error techniques to identify optimal solutions.^17^

Additionally, DL approaches can manage highly complex tasks, such as classification, segmentation, and object detection, which often demand large-scale data sets. Unlike traditional methods, DL algorithms autonomously learn the most salient data representations without requiring human intervention.^38^

## 4 3D Bioprinting of TME Models for Selected Cancer Types

To contextualize the scope of this review, this section first provides an overview of the core technologies, including a table summarizing the main 3D bioprinting techniques. Furthermore, it highlights the role of hydrogels as bioinks in 3D bioprinting for the development of in vitro tumor models. Section 4.3 focuses on the characteristics of TMEs in CRC, oral cancer, BrCa, and glioma/GBM. Understanding the technological and pathophysiological foundations of these approaches is essential for evaluating their contributions to the reconstruction of TMEs. Finally, Section 4.4 discusses current research applications of 3D bioprinting in TME modeling and includes a comprehensive table summarizing studies on selected cancer types.

### 4.1 3D Bioprinting Techniques

The most widely used 3D bioprinting methods for cancer model fabrication include Extrusion-Based Bioprinting (EBB), Jetting-Based Bioprinting (JBB), such as Drop-on-Demand (DBB), Laser-Assisted Bioprinting (LAB), and Vat Photopolymerization-Based Bioprinting (VPB), which encompass techniques such as Stereolithography (SLA), Digital Light Processing (DLP), and Two-Photon Polymerization (2PP). These methods differ significantly in resolution, printing speed, bioink compatibility, and impact on cell viability. Each technique offers distinct advantages and limitations, depending on the intended tissue structure and cell type.

A comparative overview of bioprinting modalities with characteristics, strengths, and limitations in cancer modeling is provided in Table 2.

**Table 2:** Advantages and Disadvantages of the Currently Used 3D Bioprinting Techniques

**3D bioprinting technique**		**Characterization**	**Advantages**	**Disadvantages**	**Ref.**
**Extrusion-based Bioprinting**		extrudes bioinks through a nozzle to create continuous layers, ideal for building larger structures with high cell density	ability to deposit cells at high densities	low resolution 100–1200 μm	^39,40^
			high printing speeds	high pressure and shear stress	
			broad range of bioinks	reduce cell viability during the bioprinting process	
			ease of implementation		
			availability of affordable, commercial hardware		
**Jetting-based Bioprinting**	**Drop-on-demand Bioprinting**	uses droplets of bioink deposited layer by layer, allowing for high precision and speed, suitable for printing fine structures	simplicity	restricted range of printable biomaterials, necessitating the use of low-density bioinks	^41–43^
			cost-effectiveness	nozzle clogging and inconsistent droplet size	
			rapid bioprinting speeds		
			high resolution		
			ability to incorporate a variety of biological materials		
**Laser-assisted Bioprinting**		uses laser pulses to deposit biomaterials and living cells onto a substrate with high precision, facilitating the creation of complex, layered structures	high resolution	low productivity and printing efficiency, which could be a significant drawback in organ printing	^44^
			high-throughput printing	high cost of LAB printers	
			high cell viability		
			capable of depositing high-viscosity bioinks		
			capability to achieve in situ printing		
**Vat Photopolymerization-based Bioprinting**	**Stereolithography**	uses light to selectively cure layers of a photosensitive bioresin, enabling the precise creation of highly detailed, cell-compatible 3D structures	high resolution 40–50 μm	high cost	^45,46^
			rapid printing speed	postprocessing requirements	
			material versatility	printing speed	
			localized heating is minimized during printing	material cost	
				limited number of photo-cross-linkable polymers	
	**Digital light processing**	uses a digital light projector to cure photopolymer resins layer by layer, allowing rapid and high-resolution fabrication of complex structures	faster compared to SLA	rusin waste	^47–50^
			high resolution (up to 10 μm)	material shrinkage	
			exceptional shape fidelity	long printing time	
			free free-formed technology		
	**Two-photon polymerization**	uses focused, high-intensity laser pulses to polymerize photosensitive materials at a precise focal point, enabling the creation of ultrafine, high-resolution microstructures	ultra high resolution (in nanometers)	low processing speed	^51^
			complex 3D structures	lack of commercially available two-photon polymerizable resins	
				low processing volume	
				high cost	

### 4.2 Hydrogels as Bioinks for Cancer Bioprinting

The choice of bioink is critical for maintaining cell viability and ensuring the mechanical and biochemical fidelity of printed constructs. Hydrogels are widely employed as bioinks in 3D bioprinting due to their ability to replicate the physical and biochemical properties of the native ECM. In cancer modeling, the selection of hydrogel is particularly important, as properties such as mechanical stiffness, ligand presentation, porosity, degradability, and bioactivity significantly influence tumor cell behavior and intercellular interactions within the TME.

Hydrogel selection must balance structural integrity with biological relevance. For example, stiffer matrices may simulate fibrotic tumors such as pancreatic or colorectal cancer, while softer hydrogels are suitable for brain tumor models. As emphasized by Gungor-Ozkerim et al.^52^ and Sun et al.,^21^ the development of cancer-specific bioinks remains a crucial step toward generating functionally relevant in vitro TME models.

Both natural and synthetic polymers, especially hydrogels, play crucial roles in bioprinting. Most natural polymers are water-soluble and exhibit favorable biological and physiological properties, such as flexibility comparable to soft tissues and organs, compatibility with cell encapsulation and transplantation, and ease of handling and reshaping. In contrast, the properties of synthetic polymers depend on factors such as processing conditions, molecular weight, comonomer distribution, and chain structure. Hydrogels offer a benign and stable environment that supports cell growth, migration, aggregation, proliferation, and differentiation. Their integration with 3D bioprinting technologies provides numerous advantages throughout the fabrication proces.^53^

Natural polymers used as the main components of bioinks serve the following functions: (i) providing structural support and accommodation for cells and bioactive agents; (ii) acilitating the formation of vascular, neural, and lymphatic networks as semipermeable substrates for nutrient, oxygen, metabolite, and biosignal exchange; (iii) guiding tissue and organ development in a controlled manner; and (iv) promoting tissue maturation and functional integration.

Examples of natural polymers used in 3D bioprinting are collagen, elastin, keratin, gelatin, chitin, alginate, hyaluronan, chitosan, silk, dextran, agar, and starch. Natural hydrogels, such as Matrigel, derived from Engelbreth–Holm–Swarm (EHS) mouse sarcoma, provide essential ECM components, including collagen IV and laminin, along with various growth factors.^54^ However, Matrigel’s low mechanical stiffness often limits its ability to accurately.^55^ Matrigel is frequently combined with collagen to overcome these limitations and improve mechanical properties.

Alginate is another commonly used hydrogel, valued for its tunable gelation and ease of handling. However, it lacks intrinsic cell adhesion sites, prompting strategies to enhance its biofunctionality, such as conjugating integrin-binding motifs (e.g., RGD peptides) or blending with other hydrogels like gelatin.^56,57^ Gelatin, derived from denatured type I collagen (Col1), offers excellent cell adhesion but poor mechanical stability. Chemical modifications, such as methacrylation, yield GelMA (gelatin methacryloyl), a hydrogel with tunable mechanical properties through photo-cross-linking.^58^ GelMA’s versatility and printability make it particularly well-suited for creating complex, physiologically relevant 3D tumor models.^59^ Additionally, cross-linkers like lysyl oxidase (LOX) can further modulate hydrogel stiffness, bringing the mechanical properties of bioinks closer to those of native tumor ECM.^60^

Synthetic polymers are applied for the following functions, such as (i) enhancing the mechanical properties of vascular and neural networks; (ii) providing support and protection through structural components.

Examples of synthetic polymers used in 3D bioprinting are copolymers of lactide and glycolide, poly­(glycolic acid), poly­(hydroxypropyl methacrylamide), polyurethanes, poly­(ϵ-caprolactone), polylactide, and poly­(methyl methacrylate).

Polymers used in 3D bioprinting must meet several basic criteria: nontoxicity or low toxicity, minimal immunogenicity, bioprintability, mechanical stability, biodegradability (with degradation rates aligned to tissue regeneration speed), permeability for nutrients and gases, and sterilizability.^61^

As discussed in the following sections, these hydrogels form the foundation for a wide range of cancer-specific TME models.

### 4.3 Tumor Microenvironments in Selected Cancer Types

The concept of the TME has its origins in 1863, when Rudolf Virchow first proposed a link between inflammation and cancer development, suggesting that metastasis could be explained simply by the arrest of tumor-cell emboli within the vasculature.^62^ This mechanical perspective was later challenged by Stephen Paget in 1889, who introduced his famous “seed and soil” theory, emphasizing the crucial role of the microenvironment in cancer metastasis and the close relationship between tumors and their surrounding tissue.^63^ In 1928, James Ewing contested Paget’s theory, arguing instead that mechanical forces and vascular patterns between primary tumors and secondary sites were the main determinants of metastatic organ specificity.^64^ Decades later, in the late 1970s and early 1980s, the seminal studies of Isaiah Fidler and colleagues provided definitive evidence supporting Paget’s ″seed and soil″ concept. Their research demonstrated that while tumor cells circulate through the vasculature of all organs, metastases selectively develop only in organs whose microenvironments are conducive to tumor growth. This understanding solidified the central role of TME in cancer biology and metastasis.^65^

TME is a highly complex and heterogeneous system composed of diverse cell types with tumor and stromal cells embedded in a remodeled ECM. TME may include adipocytes (notably in BrCa,^66^ liposarcomas,^67^ ovarian cancer^68^ and prostate cancer^69^), pericytes,^70^ neural elements (such as neurons in oral/head and neck cancers (HNC) and neurons and glial cells in brain cancer^71^), signaling molecules (including cytokines, chemokines^72^ and growth factors in lung cancer,^73^ BrCa,^74^ pancreatic cancer;^75^ hepatocellular carcinoma (HCC),^76^ and melanoma^77^), as well as metabolic factors (such as lactate and oxygen gradients in BrCa,^78^ CRC,^79^ lung cancer,^79^ pancreatic cancer,^80^ brain tumors,^81^ melanoma,^82^ HCC^83^) and proteolytic factors in HNC.^84^ These elements further contribute to the dynamic and complex interactions within the TME, influencing tumor progression and therapeutic resistance.

As the TME varies significantly across cancer types, reflecting tissue-specific characteristics and disease progression mechanisms, the following section briefly outlines TME features in the four cancer types analyzed in this review.

#### 4.3.1 Tumor Microenvironment in Colorectal Cancer

CRC is a complex and multifaceted disease affecting the colon and rectum, with a profound impact on gastrointestinal function and overall health. Globally, CRC ranks among the most prevalent cancers, accounting for approximately 1.9 million new cases and 900,000 deaths in 2022.^1,85^

The intricate nature of CRC presents considerable challenges in treatment due to its highly dynamic TME, which involves complex interactions among cancer cells, immune responses, stromal components, and the gut microbiota. These elements contribute to tumor heterogeneity and therapy resistance, necessitating personalized and adaptive treatment approaches.

Tumor cell-autonomous pathways, such as oncogenic signaling activation, play a significant role in CRC progression and metastasis. However, growing evidence indicates that noncell-autonomous signaling pathways within the CRC microenvironment also profoundly influence these processes.^86^ The TME is critical in the initiation, progression, and metastasis of the CRC. Figure 3 illustrates the CRC TME’s complexity, highlighting cellular elements like CAFs (cancer-associated fibroblasts), endothelial cells, macrophages, and T-cells, alongside acellular structures such as the extracellular matrix. These elements communicate through various molecular pathways that promote tumor growth, angiogenesis, immune suppression, and metastasis.

**Figure 3:**
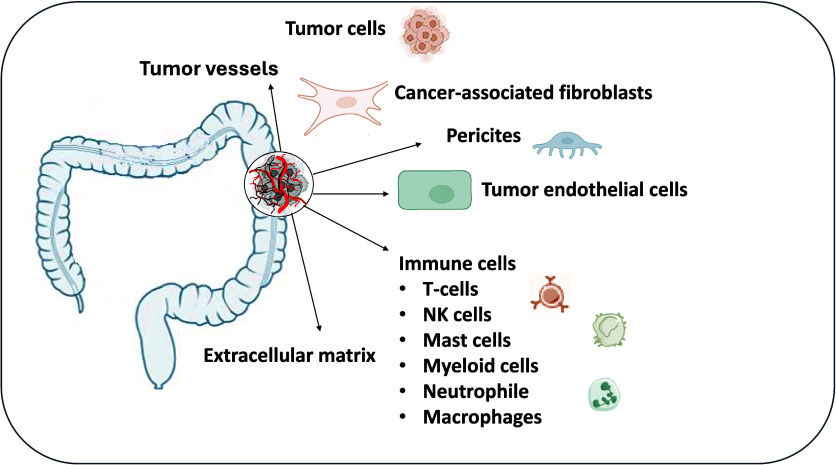
Complex microenvironment of CRC and its role in tumor progression.

CAFs play a pivotal role in shaping the TME by establishing a supportive niche. They drive tumor fibrosis through extensive ECM deposition and release various paracrine factors including cytokines, extracellular vesicles, and metabolites, which promote tumor cell proliferation, survival, and migration. Additionally, CAFs influence the metabolic and immune reprogramming of the TME, significantly affecting tumor progression and contributing to both therapeutic resistance and response modulation.^87^

The stroma compartment also plays a crucial role in regulating the metastatic potential of CRC. Studies have shown that alterations in ECM composition and tensile forces within the stromal ECM of premalignant tissues are associated with an increased likelihood of CRC progression and metastasis.^88^ Moreover, increased tumor stiffness may reduce efficient drug delivery and promote resistance.^89^

The native ECM is a sophisticated network of structural proteins, glycoproteins, glycosaminoglycans and proteoglycans that affects cell adhesion, morphology, migration and proliferation, as well as regulating tissue morphogenesis and fluid balance.^90^ In this context, ECM is recognized as a dynamic and complex structure with chemical and biophysical properties that induce different physiological and pathological cell fates. In the CRC microenvironment, the ECM plays a critical role by modulating cell signaling pathways, promoting tumor progression, supporting metastasis, and enabling cell migration.^91^

Among ECM proteins, collagens are the most abundant, with twenty-eight subtypes identified.^92^ Col1, the primary ECM component in the lamina propria of the colon mucosa, is overexpressed in CRC patients and is the most prevalent collagen subtype in connective tissues.^92–95^ Aberrant collagen deposition increased matrix density of ECM as a results result of progressively thickening, linearizing provide providing to stiffness to of Col1 fibers, observed normally as relaxed fibrils in healthy ECM. The process induces mesenchymal gene expression tumorigenesis and metastasis.^95–97^

Hypoxic areas within the TME also complicate treatment as hypoxia induces mechanisms of chemotherapeutic resistance and poor response to therapy. Integrating these factors into long-term 3D bioprinted models would provide a powerful tool for monitoring tumor evolution under various therapeutic conditions. Such models could simulate hypoxic zones and necrotic regions, enabling a side-by-side comparison of patient-specific tumor dynamics and offering insights into adaptive responses to treatment.^98,99^

Recent studies have also emphasized the unique role of the gut microbiota in CRC carcinogenesis^100^.^101^ The gut microbiota (*Fusobacterium nucleatum*, *Helicobacter pylori*, *Streptococcus bovis*, *Streptococcus Gallolyticus*, certain strains of *Escherichia coli*, and producing enterotoxins *Bacteroides fragilis* strains) can drive carcinogenesis through metabolites and signaling molecules that affect responses in both host epithelial and immune cells.^102,103^ Close interactions between the intestinal microbiota and the TME may directly influence the effectiveness of chemotherapy.^104,105^ Currently, there are several genera of bacteria that accumulate preferentially in tumors. Their favorable tumor penetration ability is used in cancer therapy.^104,106,107^

#### 4.3.2 Tumor Microenvironment in Head and Neck Cancers

This section focuses on head and neck cancers (HNCs), a heterogeneous group of malignancies that include oral, pharyngeal, and laryngeal cancers. Due to the scarcity of high-resolution 3D bioprinting studies in this group, available findings related to oral cancer, a major HNC subtype, are presented as representative examples.

Oral cancer is an intricate and multifaceted disease impacting anatomical structures in the head and neck area, such as the tongue, pharynx, mucous membranes, and bones.^108^ Globally, it ranks as the sixth most prevalent form of cancer,^109^ with approximately 380,000 new cases and 180,000 deaths in 2020,^110^ placing a substantial strain on global healthcare systems.

The implications of oral cancer on oral health extend to critical aspects such as communication, nutrition, self-esteem, and social interactions, significantly influencing the overall well-being of individuals. However, the intricacy of oral cancer poses challenges in treatment as a result of its intricate microenvironment, necessitating tailored therapeutic strategies.

The oral cancer TME is composed of a variety of cellular and noncellular elements that interact dynamically to influence tumor behavior (Figure 4). Among the key cellular components are CAFs,^111^ tumor-infiltrating lymphocytes, macrophages, endothelial cells, and neural elements such as peripheral neurons and glial cells, which are particularly relevant in head and neck cancers.^112^ These cells contribute to immune evasion, tumor progression, and metastasis through the release of cytokines, chemokines, and extracellular vesicles.

**Figure 4:**
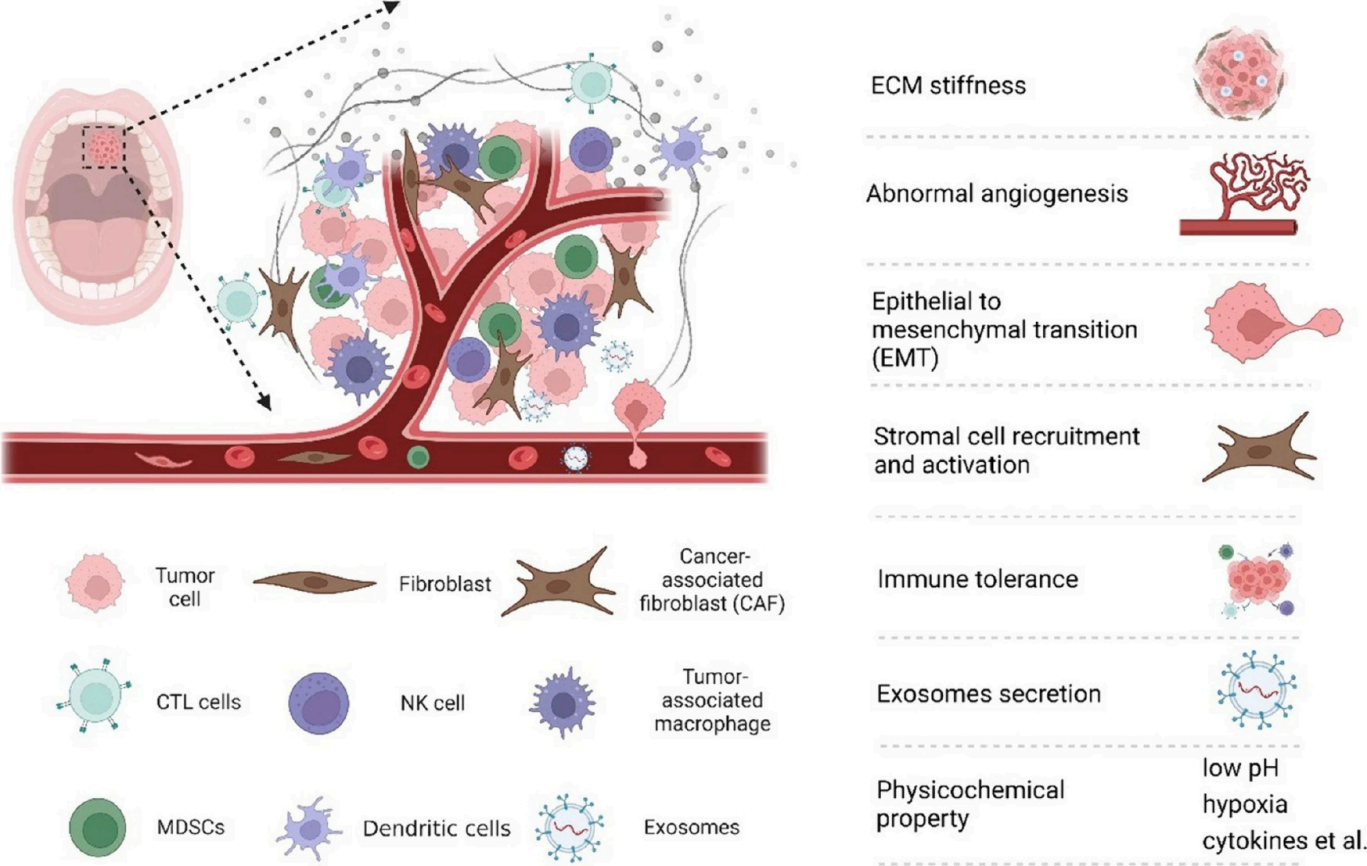
Schematic diagram of the TME of oral cancer. Reproduced from ref 112. Available under a CC-BY 4.0 license. Copyright 2022 Liu et al.

A crucial feature of the TME in oral cancer is remodeling of the ECM. The ECM undergoes substantial biochemical and biomechanical changes, including increased stiffness and altered collagen composition, which facilitate tumor cell migration and invasion. Matrix metalloproteinases (MMPs), particularly MMP-2 and MMP-9, are overexpressed in oral squamous cell carcinoma and play a significant role in ECM degradation and metastatic spread.^113,114^

Hypoxia is another hallmark of the oral cancer TME, especially in poorly vascularized regions. It contributes to treatment resistance, epithelial-mesenchymal transition (EMT), and a more aggressive tumor phenotype. The hypoxic microenvironment activates hypoxia-inducible factors (HIFs), which regulate genes involved in angiogenesis, metabolism, and survival pathways.^115^

Recent studies have also highlighted the involvement of the oral microbiome in carcinogenesis. Specific bacterial species, such as *Porphyromonas gingivalis*,^116^ *Fusobacterium nucleatum*,^117^ and *Treponema denticola*,^118^ have been implicated in tumor-promoting inflammation and modulation of immune responses within the TME.^119,120^ These microbes may facilitate tumor progression through chronic inflammation, DNA damage, and suppression of antitumor immunity.^119^

#### 4.3.3 Tumor Microenvironment in Breast Cancer

According to the GLOBOCAN database, BrCa was responsible for approximately 665,000 deaths globally in 2022.^1^ With the highest number of diagnoses over the past five years, BrCa has become the most prevalent cancer worldwide.^121^

One of the greatest clinical challenges in BrCa is its high degree of heterogeneity. This heterogeneity occurs both between patients (intertumoral) and within individual tumors (intratumoral), and is considered a hallmark of malignancy.^122^ It contributes to variable treatment responses, therapy resistance, and disease progression, complicating the design of effective, one-size-fits-all therapeutic strategies.

BrCa heterogeneity is largely driven by complex interactions between tumor cells and their surrounding microenvironment. The breast TME consists of various stromal and immune cells, ECM components, signaling molecules, and adipose tissue (Figure 5). A key stromal component in the breast TME is adipose-derived mesenchymal stem cells (ADMSCs), which are abundant in mammary adipose tissue and have been shown to play a dual role in cancer biology.^123,124^

**Figure 5:**
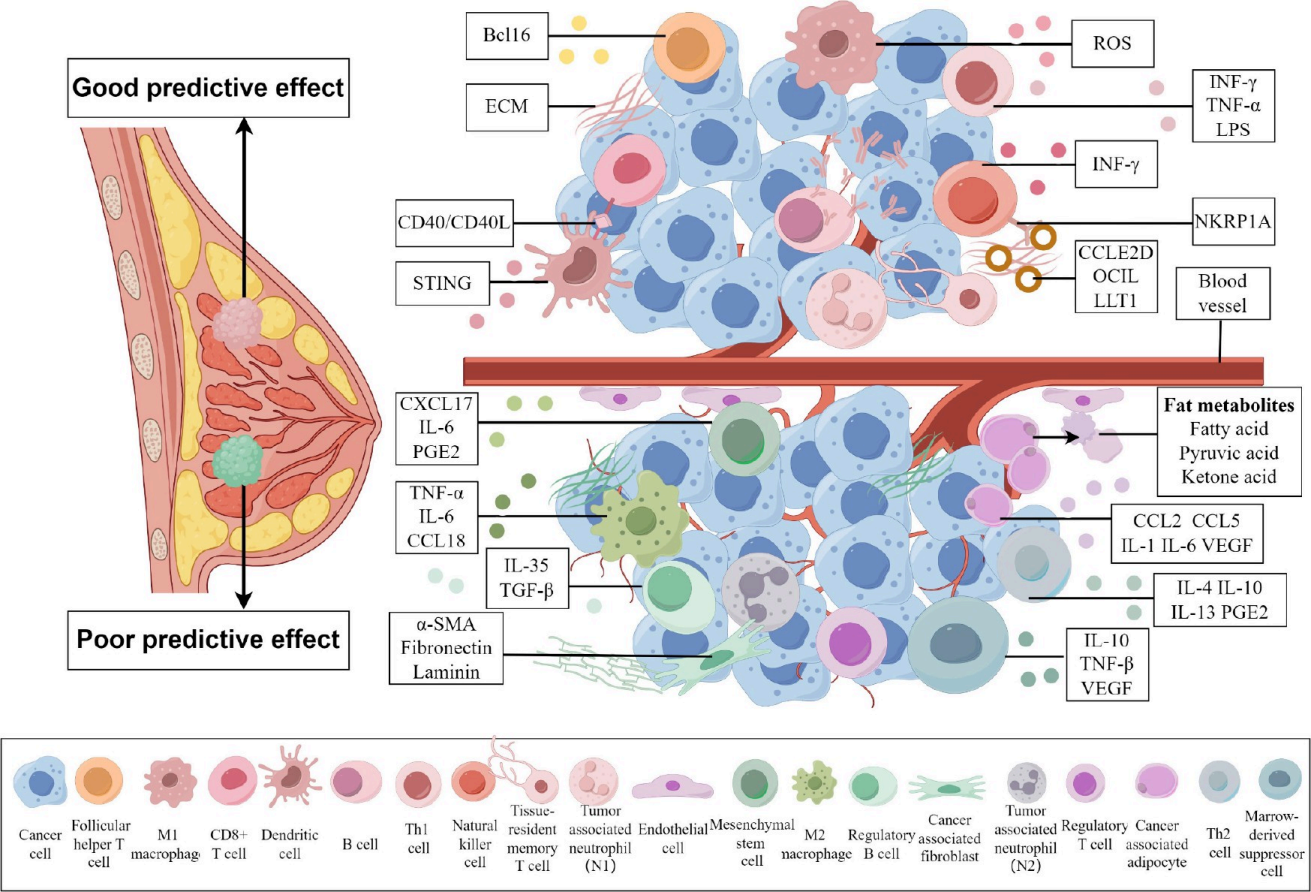
Schematic diagram of the TME of BrCa. Reproduced from ref 125. Available under a CC-BY 4.0 license. Copyright 2024 Dou et al.

The contribution of ADMSCs to BrCa progression remains a subject of ongoing debate. Some studies suggest that they promote tumor growth, invasion, and metastasis by releasing cytokines, chemokines, and extracellular vesicles that enhance cancer cell proliferation and survival. Other studies, however, report antitumor effects under specific conditions.^126^ This ambiguity highlights the need for more physiologically relevant models to investigate the multifaceted role of ADMSCs in the breast TME.

#### 4.3.4 Tumor Microenvironment in Glioma Cancer

Gliomas are among the most common adult-type primary central nervous system (CNS) tumors, with GBM representing the most aggressive and lethal form. Despite advancements in diagnostics and therapy, the prognosis for GBM remains poor, with a five-year relative survival rate increasing only modestly, from 23% in the late 1970s to 36% between 2009 and 2015.^127^ The complexity of brain tumors, combined with their location and limitations of current treatment modalities, underscores the need for a deeper understanding of the glioma TME.

The TME of glioma is highly specialized and contributes significantly to tumor heterogeneity, progression, and therapeutic resistance. It comprises a diverse array of cellular elements, including endothelial cells, neurons, astrocytes, oligodendrocytes, and resident immune cells (e.g., microglia; Figure 6). These components interact with a noncellular matrix enriched in signaling molecules, extracellular vesicles, and a unique ECM. Unlike many other solid tumors, the ECM in glioma is notably deficient in collagens but rich in hyaluronic acid (HA),^128^ glycosaminoglycans,^129^ proteoglycans,^130^ and glycoproteins.^131^ Enzymes responsible for ECM remodeling further modulate this specialized microenvironment, influencing cell behavior and tumor invasiveness.

**Figure 6:**
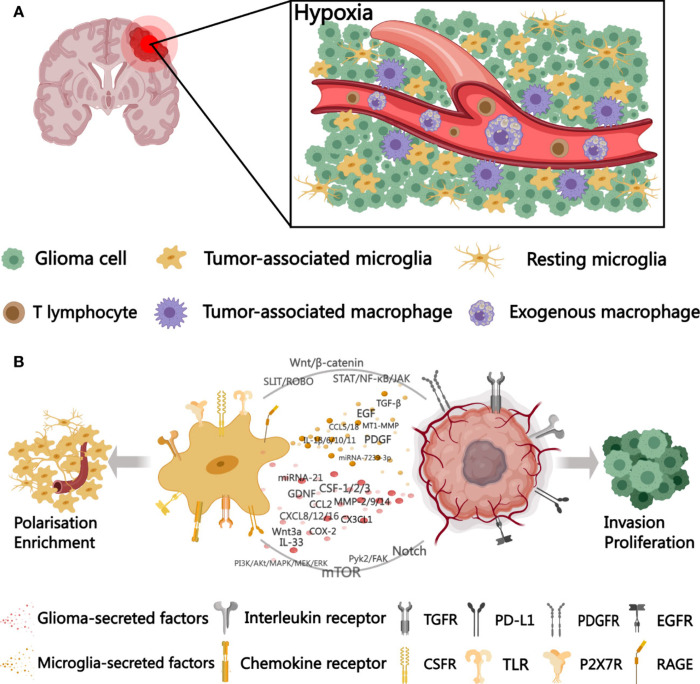
Schematic diagram of the microglia-glioma cell interactions in the TME. Reproduced from ref 134. Available under a CC-BY 4.0 license. Copyright 2023 Tao et al.

Hypoxia is another defining feature of the glioma TME, contributing to the formation of necrotic cores that are characteristic of high-grade tumors like GBM. The resulting hypoxic gradients promote stemness, metabolic adaptation, angiogenesis, and resistance to conventional therapies.^132^ These hypoxic zones also influence the spatial organization and phenotype of glioma stem-like cells, which are key drivers of tumor recurrence.^133^

A comprehensive understanding of the glioma TME and the ability to replicate its features in vitro is essential for the development of novel, targeted therapeutic strategies and for improving patient outcomes in this highly lethal cancer.

### 4.4 Applications of 3D Bioprinting in TME Modeling

Traditional 2D in vitro cancer models fall short of capturing this complexity, as they lack the cellular diversity and spatial architecture found in vivo (Figure 7). Such models fail to replicate the multifaceted interactions between tumor and stromal components, which limits their predictive value for clinical outcomes.

**Figure 7:**
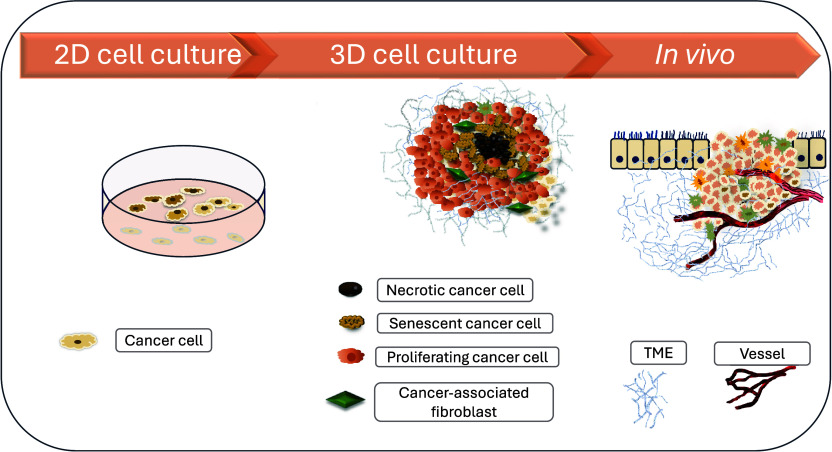
Tumor environment under various culture conditions: 2D, 3D, and in vivo.

A growing body of research has focused on the use of 3D bioprinting to construct physiologically relevant TME models. This technology offers a promising platform for incorporating ECM-mimicking hydrogels, cocultures of tumor and stromal cells, and potentially even microbiome components.

Table 3 provides a detailed overview of the publications included in this scoping review on the topic of 3D bioprinting of TME models for selected cancer types, retrieved from PubMed and published between January 2020 and June 2025 in English. Each entry details the authorship, year of publication, country of origin, methodological design, experimental population, and key findings related to the use of 3D bioprinting for TME modeling. From an initial pool of 63 identified articles, 44 were manually selected based on relevance and predefined inclusion criteria. These include 3 studies focused on CRC, 2 on oral cancer, 30 on breast cancer, and 9 on glioma (including GBM), representing the four cancer types defined within the scope of this review.

**Table 3:** Summary of Publications on 3D Bioprinting Models of the TME for Selected Cancer Types, Including Authors, Year of Publication, Country, Methodological Design, Study Population, and Main Findings

**Cancer type**	**Authors**	**Year of publication, country**	**Methodological design**	**Population**	**Main findings**	**Ref.**
**Colorectal cancer**	Zhou H. et al.	2024, China	In vitro; single-cell/spatial analysis	Mucinous colorectal adenocarcinoma	MUC1-mediated crosstalk in CRC TME.	^135^
	Chen H. et al.	2020, China	In vitro; CRC tumor tissue model 3D printed	Colorectal tumor model	Colorectal TME model with therapeutic applications.	^136^
	Cadamuro F. et al.	2023, Italy	In vitro; hyaluronic acid + signaling glycans	CRC bioprinted model	Hyaluronic bioinks reliably recreated CRC TME for drug testing.	^137^
**Oral cancer**	Almela T. et al.	2021, UK	In vitro; 3D bioprinting of oral cancer	Oral cancer cells	Successfully reproduced histological TME.	^138^
	Siquara da Rocha LO. et al.	2023, Brazil	In vitro; cell-in-cell structures model	Oral squamous carcinoma cells	Explored cell-in-cell phenomena in oral cancer.	^139^
**Breast cancer**	Desigaux T. et al.	2024, France	In vitro; 3D bioprinting	Breast cancer model (stromal fibroblasts + tumor cells)	Stroma modulated ECM and influenced radiosensitivity.	^140^
	González-Callejo P. et al.	2023, Spain	In vitro; tumor–stroma bioprinting	Breast cancer + stroma	Enabled preclinical drug testing with faithful tumor–stroma interactions.	^141^
	Yuan T. et al.	2024, USA	In vitro; spatially defined breast tumor	Breast tumor cells + stromal spheroids	Model demonstrated drug resistance and tumor heterogeneity.	^142^
	González-Callejo P. et al.	2024, Spain	In vitro; triple-negative breast cancer model	TNBC cells + stroma	Assessed targeted therapies with tumor–stroma interplay.	^143^
	Nanou A. et al.	2022, Netherlands	In vitro; additive-manufactured scaffolds	Metastatic breast cancer cells	Model supported cancer cell migration.	^144^
	Hong S. and Song J. M.	2022, Korea	In vitro; bioprinted breast cancer spheroids	Breast cancer spheroids	Breast cancer spheroids	^145^
	Bojin F. et al.	2021, Romania	In vitro; TME tissue model bioprinting	Multitissue tumor models	Platform for TME mimicry.	^146^
	Dey M. et al.	2021, USA	In vitro; angiogenesis and invasion model	Breast cancer neurovascular unit	Analyzed tumor angiogenesis/invasion.	^147^
	Lee G. et al.	2023, Korea	In vitro; microfluidic vascularized array	Multicomposition tumor array	High-throughput drug evaluation	^148^
	Horder H. et al.	2021, Germany	In vitro; adipose stromal cell spheroids	Breast cancer + adipose stromal cells	Tumor cell migration depends on stromal spheroids.	^149^
	Moghimi N. et al.	2023, Canada	In vitro; tumor-on-chip for heterogeneity	Breast cancer + vascularization	Controlled heterogeneity in coculture.	^150^
	Dey M. et al.	2022, USA	In vitro; CAR-T + vascularized model	Breast cancer + CAR-T + vascular support	Platform for immunotherapy and chemo testing.	^151^
	Dey M. et al.	2022, USA	In vitro; MAIT receptor T cell model	Breast cancer + engineered T cells	Evaluated cytotoxic response.	^152^
	Redmond J. et al.	2021, Ireland	In vitro; collagen-based biofabrication	Breast cancer scaffold model	Collagen scaffold techniques in breast cancer.	^153^
	Saemundsson SA. et al.	2023, USA	In vitro; DNA-directed spheroid coculture	Mixed cell spheroids	Controlled cell organization via DNA interactions.	^154^
	Kort-Mascort J. et al.	2023, USA	In vitro; ECM-based bioink tumor model	Breast cancer spheroids	Progressive remodeling in ECM-based printed models.	^155^
	Gebeyehu A. et al.	2021, USA	In vitro; polysaccharide hydrogel models	Tumor cells (MDA-MB-231 WT)	Tested chemotherapeutic responses.	^156^
	Suarez-Martinez AD. et al.	2021, USA	In vitro; bioprinting on live tissue	Multiple tumor cell types	Investigated dynamic cancer cell behavior on live tissue.	^157^
	Horder H. et al.	2024, Germany	In vitro; adipose-stromal spheroid model	Breast cancer + ASC spheroids	Migration dependent on adjacent spheroids.	^158^
	Chen Y. et al.	2025, USA	In vitro; fibroblast-mediated TME	Tumor–stroma interactions	Studied tumor–stroma response and screening.	^159^
	Boroojeni F. R. et al.	2024, Sweden	In vitro; proteolytic remodeling model	Tumor microenvironment	Modeled proteolytic remodeling in TME.	^160^
	Han J. et al.	2025, China	In vitro; patient-derived organoid arrays	Breast cancer patient organoids	Captured intrinsic and extrinsic tumor characteristics.	^161^
	Liu T. et al.	2021, USA	In vitro; lymphangiogenesis model	Breast tumor tissue model	Modeled lymphangiogenesis.	^162^
	Bjerring JS. et al.	2025, USA	In vitro; mitochondrial transfer model	Breast tumoroids	Mitochondrial transfer alters cell fate	^163^
	Lee G. et al.	2024, Korea	In vitro; multicomposition tumor array	Breast tumor array	Multivariable drug efficacy testing.	^164^
	Breideband L. et al.	2025, Germany	In vitro; gravity and stiffness modulation	Breast cancer spheroids	Gravitational force and stiffness affected invasiveness.	^165^
	Mei X. et al.	2025, Mexico	In vitro; animal patient-derived model	Breast cancer organoids	Anticancer drug screening platform.	^166^
	Ferreira LP. et al.	2025, Portugal	In vitro; fibrous decellularized model	Breast tumor stroma	Fibrous decellularized models for TME.	^167^
	Tang M. et al.	2020, USA	In vitro; glioblastoma microenvironment	In vitro; glioblastoma microenvironment	In vitro; glioblastoma microenvironment	^168^
	Chaji S. et al.	2020, USA	In vitro; adipocyte–cancer interactions	Breast adipocyte + cancer cells	Modeled adipocyte-cancer interactions.	^169^
**Glioma (including glioblastoma, GBM)**	Wang X. et al.	2021, China	In vitro; glioma environment with vasculature	Glioma cells + vasculature	Effectively recreated glioma angiogenesis in 3D.	^170^
	Tung YT. et al.	2024, USA	In vitro; neurovascular glioblastoma model	Glioblastoma + vascular cells	Model captured tumor growth and neurovascular interactions.	^171^
	Oliver L. et al.	2024, France	In vitro; coculture of MSCs + glioblastoma	MSC + glioblastoma	MSCs influenced glioblastoma gene expression in coculture.	^172^
	Dai X. et al.	2022, USA	In vitro; glioma stem + MSC coculture	Glioma stem cells + MSC	Fusion promotes malignancy.	^173^
	Tang M. et al.	2021, USA	In vitro; rapid glioblastoma bioprinting	Glioblastoma tumor heterogeneity	Mimicked biophysical heterogeneity.	^174^
	Liu D. et al.	2024, USA	In vitro; radiotherapy-resistant glioma	Glioma cells + ITGA2/p-AKT pathway	Identified pathway in 3D bioprinting context.	^175^
	Zielniok K. et al.	2024, Poland	In vitro; glioma + MSC coculture	Glioblastoma + MSC	Perivascular niche chemokine influences.	^176^
	Wang X. et al.	2023, China	In vitro; glioma stem cell vascular study	Glioma stem cells + environment	Vascularization ability in microenvironments.	^177^
	Chehri B. et al.	2024, USA	In vitro; drug-delivered hydrogel mesh	Glioblastoma tumor model	3D-printed hydrogel mesh for localized drug release.	^178^

From the broader pool of studies identified and analyzed, representative examples were selected for detailed discussion based on their relevance, methodological novelty, and clear demonstration of key trends in 3D bioprinting of TME models. The selected works reflect the diversity of approaches across bioinks, tumor microenvironment features, and cancer types and are intended to illustrate typical strategies rather than provide an exhaustive analysis.

In CRC modeling, 3D bioprinting strategies aim to replicate not only the cellular composition but also the structural and mechanical properties of the TME, including glandular architecture and epithelial barriers. These features are critical for accurately reproducing the complex interactions among tumor cells, stromal components, and the ECM, which cannot be effectively captured in traditional 2D cultures. Col1-based hydrogels are frequently employed due to their biomimetic properties and ability to support tumor-stroma interactions. Chen et al. developed a 3D-printed CRC model incorporating CRC cells, CAFs, and tumor-associated endothelial cells (TECs) within a collagen scaffold. This model demonstrated enhanced gland-like structures and epithelial integrity, leading to improved physiological relevance and drug response prediction compared to 2D cultures (Figure 8).^136^

**Figure 8:**
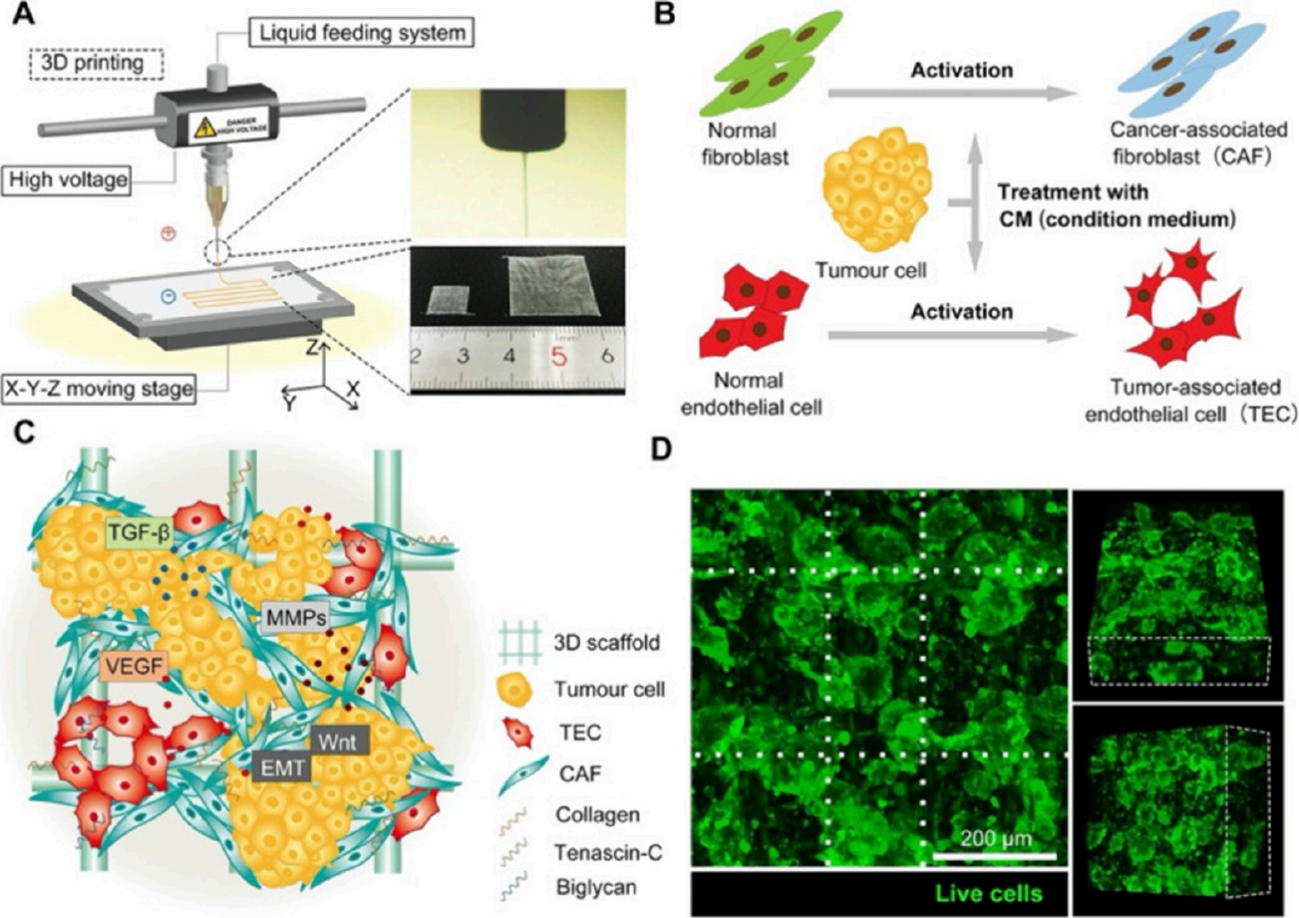
Construction and characterization of the *in vitro* 3D tumor tissue. (A) Schematic illustration of the E-jet 3D printing device. (B) Flowchart of cell activation pathways. (C) Illustration of the 3D tumor tissue. (D) Fluorescence images of the tumor tissue (green = live cells, scale bar = 200 μm). White dotted lines indicate the centers of the scaffold fibers. Reproduced from ref 136. Available under a CC-BY 4.0 license. Copyright 2020 Chen et al.

Expanding on the importance of ECM composition, Cadamuro et al. further refined CRC models by utilizing HA-based bioinks enriched with signaling glycans, capturing key features of tumor-stroma interactions. Their constructs better mimicked the biochemical composition of the native TME and facilitated the formation of epithelial barriers crucial for tumor modeling.^137^

In oral squamous cell carcinoma (OSCC), 3D bioprinting enables the construction of in vitro models that capture complex cellular interactions and ECM remodeling characteristic of the oral TME. Almela et al. developed a 3D bioprinted OSCC model emphasizing the inclusion of CAFs to replicate tumor–stroma interactions and desmoplastic ECM remodeling. These elements are pivotal for accurately mimicking the invasive behavior of oral tumors.^138^ Moreover, emerging models increasingly recognize the role of neural components and neuron–tumor interactions in OSCC progression.

Siquara da Rocha et al. highlighted the significance of cell–cell interactions through their mapping of cell-in-cell structures within OSCC tissues, shedding light on tumor aggressiveness and immune evasion mechanisms, and underscoring the need for 3D models capable of capturing such complexities.^139^

BrCa remains the most frequently studied tumor type within the field of 3D bioprinting for TME modeling. This dominance reflects both the well-characterized cellular and the stromal components of the breast microenvironment. Additionally, the relative ease of integrating BrCa models into microfluidic and tumor-on-a-chip systems has further accelerated research efforts in this area.

Desigaux et al. developed a 3D bioprinted BrCa model incorporating fibroblasts to investigate ECM remodeling and its influence on radiosensitivity. Their findings underscored how stromal elements shape the biomechanical properties of the TME, directly impacting tumor behavior and treatment outcomes.^140^ González-Callejo et al. advanced this work by including adipocytes in their constructs, reflecting the unique role of adipose tissue in BrCa progression and therapeutic response.^141^

Yuan et al. introduced spatial heterogeneity into 3D bioprinted BrCa models through distinct bioink formulations, allowing for the recreation of localized variations in the ECM and cell populations. This approach enabled the study of intratumoral differences in drug resistance mechanisms and microenvironmental influence on tumor progression.^142^

Additionally, Moghimi et al. contributed to this growing body of research with a bioprinted tumor-on-chip platform designed to control tumor heterogeneity through coculture systems. Their model demonstrated how precisely engineered spatial arrangements of tumor and stromal cells within microfluidic devices could replicate the complexity of the BrCa microenvironment and improve the predictability of drug testing platforms (Figure 9).^150^

**Figure 9:**
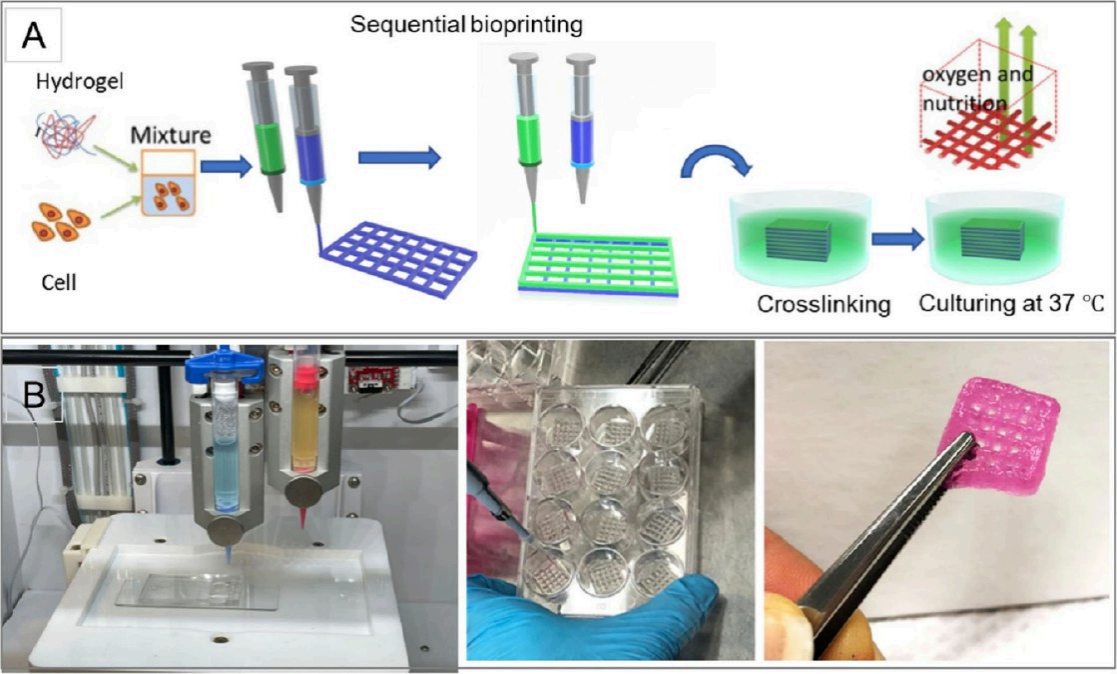
Application of AI to 3D bioprinting: a clinical focus. (A) Schematic illustration of the biofabrication process. (B) The printer and bioprinted constructs (food coloring used for the purpose of illustration). Reproduced from ref 150. Available under a CC-BY 4.0 license. Copyright 2023 Moghimi et al.

GBM models present unique challenges due to the tumor’s neurovascular context, hypoxia, and interactions with stromal components such as mesenchymal stem cells (MSCs). Wang et al. developed vascularized glioma models using 3D bioprinting to recreate tumor-induced angiogenesis and tumor–vascular interactions.^170^ Tung et al. expanded on this by constructing a human neurovascular unit model that integrated endothelial cells, pericytes, and astrocytes to simulate the hypoxic brain microenvironment and its impact on GBM growth and therapy resistance.^171^

Oliver et al. leveraged 3D bioprinting for coculture systems involving MSCs and glioma cells, revealing transcriptional changes associated with immune modulation and tumor progression, highlighting the dynamic stromal - tumor crosstalk within GBM microenvironments.^172^

Across the selected cancer types, 3D bioprinting approaches reveal both shared strategies and disease-specific adaptations. In CRC and OSCC, emphasis is placed on replicating epithelial structures, ECM composition, and stromal crosstalk. In BrCa, the inclusion of adipocytes and the modeling of spatial heterogeneity align with the unique biology of mammary tissues. Glioma models, in turn, prioritize neurovascular components, hypoxia, and MSC interactions that are reflective of the brain microenvironment. Collectively, these studies underscore the flexibility of 3D bioprinting in capturing the complexity of diverse TMEs, advancing both mechanistic studies and preclinical testing platforms.

Table 4 summarizes the key studies discussed in this section, illustrating representative approaches to 3D bioprinting of TME models across selected cancer types. This synthesis highlights key differences in bioink composition, TME features modeled, and specific research focuses, thereby facilitating a clearer understanding of current trends and priorities within this rapidly evolving field.

**Table 4:** Comparative Overview of 3D Bioprinting Strategies for Modeling Tumor Microenvironments Across Selected Cancer Types, Summarizing Bioinks, Modeled Features, Representative Studies, and Research Applications

**Cancer Type**	**Key Bioinks/Materials**	**Main TME Features Modeled**	**Primary Research Focus**
**Colorectal cancer**	Col1, HA	Glandular structures, epithelial barriers, tumor–stroma interactions	ECM remodeling, epithelial integrity, drug testing
**Oral cancer**	Col1, fibroblast-rich bioinks	ECM remodeling, fibroblast activation, potential neuro–tumor crosstalk	Invasiveness, immune evasion, stroma–tumor interactions
**Breast Cancer**	Col1, HA, adipocytes, spatial bioinks	ECM composition, adipose tissue, intratumoral heterogeneity	Drug resistance, radiosensitivity, tumor-on-chip platforms
**Glioma/GMB**	Col1, neurovascular unit components, MSC cocultures	Vascularization, hypoxia, neurovascular niche, MSC interactions	Angiogenesis, hypoxia-driven resistance, stromal crosstalk

This comparative overview highlights both the diversity and the common challenges in the 3D bioprinting of TME models across different cancer types. These findings underline the need for further refinement of bioprinting strategies, particularly in achieving physiological relevance, reproducibility, and scalability. In this context, the integration of AI technologies offers significant potential to optimize bioprinting processes, material selection, and model validation.

The following sections explore the emerging role of AI in enhancing each stage of 3D bioprinting, from preprinting design through printing control to postprinting analysis. Section 5 focuses on AI applications in 3D bioprinting across various biomedical contexts, while Section 6 narrows the discussion to the integration of AI and 3D bioprinting specifically within the scope of TME modeling.

## 5 AI in 3D Bioprinting: From Preprinting to Postprinting Stages

Although 3D bioprinting holds great potential for advancing tissue engineering and regenerative medicine, current technologies still face significant challenges in achieving reproducible, scalable, and physiologically relevant constructs. Issues such as optimizing bioink formulations, refining complex designs, ensuring precision during the printing process, and validating the biological functionality of printed models remain critical. In response, AI has emerged as a transformative tool across all stages of the 3D bioprinting workflow, enhancing the efficiency, accuracy, and adaptability.

The integration of AI into 3D bioprinting processes builds upon early foundational works that emphasized the potential of ML, DL, and digital twins in advancing fabrication precision and biological maturation of tissue constructs.^179,180^ These studies laid the essential groundwork for leveraging AI to enhance both the technical and biological aspects of bioprinting, inspiring subsequent research on data-driven optimization in this field.

This section reviews AI applications in 3D bioprinting without limiting the scope to oncology or TME models. Instead, it draws upon diverse biomedical and engineering contexts, including AI-enhanced bioink optimization, digital modeling, process monitoring, and postprinting evaluation. This broader approach provides essential insights into how AI contributes to the refinement of bioprinting technologies, laying a technological foundation for understanding its future applications in cancer-specific TME modeling (Section 6).

AI enhances bioprinting capabilities by addressing key challenges and driving innovation in process optimization and the design of intricate tissue structures. Through predictive modeling, AI algorithms improve the accuracy of bioprinting processes, forecasting mechanical properties, tissue development outcomes, and cellular behaviors. This facilitates not only precision and reproducibility but also real-time adjustments through advanced data analysis. Furthermore, AI-driven approaches provide insights into how cells and tissues respond to varying bioink compositions and process conditions, advancing control over fabrication outcomes.

The studies included in this section highlight how AI and ML are applied to optimize 3D bioprinting processes, materials, and postprocessing strategies.

Table 5 summarizes the publications included in this section, providing an overview of AI applications in 3D bioprinting across diverse biomedical contexts. The synthesis highlights the variety of ML algorithms employed, their country of origin, and their application domains, illustrating how AI supports bioink optimization, process control, structural design, and postprinting evaluation. By consolidating these studies, the table provides a structured comparison that clarifies the current scope of AI integration in 3D bioprinting and underscores the field’s evolving research priorities.

**Table 5:** Summary of Publications on AI Applications in 3D Bioprinting, Including Authors, Year of Publication, Country, ML Algorithm, and Its Application within the Context of 3D Bioprinting

**Stage**	**Authors**	**Year of publication, country**	**ML algorithm**	**Application**	**Ref.**
**Preprinting**	Sang S. et al.	2023, China	AI-assisted bioink formulation	MSC-laden bioink optimization.	^181^
	Dai Y. et al.	2025, Singapore	AI-assisted modeling	Soft tissue oral bioprinting design, predictive modeling.	^182^
	Bracco F. et al.	2025, Italy	Transfer learning	Optimizing preprinting parameters and improving printing accuracy.	^183^
	Lee J. et al.	2020, Korea	ML regression	Predicting bioink properties (modulus, yield stress)	^184^
	Sarah R. et al.	2025, Germany	Neural Networks	Predicting bioink mechanical properties	^185^
	Cadamuro F. et al.	2025, Italy	AI-assisted algorithms (unspecified)	ECM-mimetic hydrogel formulation prediction.	^186^
	Ege D. et al.	2024, Germany	XGBoost	Predicting hydrogel stiffness (ADA-GEL based bioinks).	^187^
	Reina-Romo E. et al.	2021, Spain	Optimization algorithms	Nozzle design optimization for extrusion bioprinting.	^188^
	Valenzuela-Reyes M. B. et al.	2025, Mexico	AI-assisted optimization algorithms	Scaffold design optimization via computational models.	^189^
**Printing**	Jin Z. et al.	2023, USA	Deep Neural Networks	Real-time anomaly detection during EBB.	^190^
	Kang R. et al.	2024, China	AI-based control algorithms	Microvalve-controlled extrusion bioprinting system.	^191^
	Tian S. et al.	2021, USA	Linear Regression	Predicting printing outcomes, including cell viability and filament diameter, for cell-containing alginate and gelatin composite bioinks	^192^
			Logistic Regression		
			Random Forest		
			Classification		
			Random Forest		
			Regression		
			Support Vector Machines		
			Support Vector		
			Regression		
	Guan J. et al.	2021, USA	Deep Leraning	Compensating light scattering effects in stereolithography bioprinting.	^193^
	Shin J. et al.	2025, Korea	Machine Learning (unspecified)	Optimization of high-throughput droplet bioprinting.	^194^
	Mohammadrezaei D. et al.	2024, Canada	Bayesian optimization	Process parameter optimization for extrusion bioprinting.	^195^
**Postprinting**	Andrews A. E. et al.	2023, UK	Convolutional Neural Network	Predicting mechanical properties of lab-grown tissues.	^196^
	Sheikh Z. A. et al.	2025, USA	Convolutional Neural Network	Predicting spheroid viability; quality control automation.	^197^
	Sampaio da Silva et al.	2024, Switzerland	Convolutional Neural Network	High-throughput spheroid sorting for quality control.	^198^

### 5.1 AI in Preprinting Processes

AI plays a crucial role in the early stages of bioprinting by optimizing bioink formulations, modeling tissue constructs, and refining the printing parameters. A key challenge in bioprinting is ensuring the printability of bioinks, which is largely influenced by properties, such as viscosity, yield stress, and shear-thinning behavior. These parameters directly affect the extrusion process, structural fidelity, and mechanical stability of the printed constructs. AI-based approaches offer significant potential to address these challenges through predictive modeling, enabling researchers to simulate mechanical properties, optimize bioink compositions, and enhance structural designs before fabrication.

For example, Sang et al. developed AI-assisted methods to optimize MSC-laden bioinks for cartilage regeneration.^181^ Similarly, Dai et al. integrated AI into the design of oral soft tissue constructs to improve predictive modeling and personalization.^182^ Bracco et al. utilized transfer learning to enhance the accuracy of preprinting parameter predictions, increasing efficiency and reducing experimental workload.^183^

AI is also applied to refine the physical aspects of bioprinting hardware. Reina-Romo et al. optimized nozzle designs for extrusion-based bioprinting through computational algorithms, aiming to improve printing stability.^188^ Valenzuela-Reyes et al. employed computational modeling to enhance scaffold design, contributing to more precise and functional constructs.^189^ Furthermore, studies by Sarah et al.^185^ and Ege et al.^187^ demonstrated the use of machine learning to predict the mechanical performance of novel bioinks, such as ADA-GEL hydrogels, while Cadamuro et al.^186^ utilized AI-assisted algorithms to forecast ECM-mimetic hydrogel formulations.

Through these advancements, AI-driven strategies in the preprinting stage enhance the predictability and reproducibility of bioprinting processes, offering considerable improvements in efficiency, construct quality, and the rational design of bioinks tailored to specific applications.

### 5.2 AI in Printing Processes

During the active printing phase, AI plays a vital role in enhancing precision and control. Real-time process monitoring, anomaly detection, and adaptive feedback systems are key applications. These AI technologies support adjustments in extrusion parameters, droplet formation, and exposure settings, which directly impact the structural fidelity and biological performance of printed constructs.

Several studies have demonstrated the practical benefits of AI in this phase. Jin et al. employed deep neural networks for real-time anomaly detection during extrusion-based bioprinting, allowing for immediate corrective actions.^190^ Kang et al. integrated AI-based control algorithms within microvalve-regulated systems, optimizing extrusion precision.^191^ Guan et al. applied deep learning to correct light-scattering effects during stereolithography, improving printing resolution and consistency.^193^ Mohammadrezaei et al. used Bayesian optimization to fine-tune extrusion parameters, directly enhancing cell viability and print fidelity.^195^

Additionally, Tian et al. utilized ensemble machine learning models to predict key printing outcomes such as cell viability and filament diameter, enabling more accurate calibration of complex bioink systems.^192^ Shin et al. optimized high-throughput droplet bioprinting through machine learning, streamlining processes for scalable tissue engineering applications.^194^

Collectively, these studies illustrate how AI integration in the printing phase leads to greater reproducibility, higher-quality outputs, and enhanced efficiency.

### 5.3 AI in Postprinting Processes

In postprinting stages, AI significantly contributes to evaluating the quality and functionality of bioprinted constructs. The use of convolutional neural networks (CNNs) is particularly prominent in automating quality control, predicting mechanical properties, and analyzing the viability of cell-laden constructs. These technologies enable noninvasive assessments and high-throughput analysis, crucial for standardizing bioprinted products.

Andrews et al. leveraged CNNs to predict mechanical properties of bioprinted tissues, providing rapid insights into construct integrity.^196^ Sheikh et al. developed CNN-based methods to automate quality control by predicting spheroid viability, ensuring consistency in bioprinted models.^197^ Sampaio da Silva et al. created high-throughput sorting systems for evaluating 3D spheroids, facilitating quality control in large-scale applications.^198^

These AI-driven approaches in postprinting stages advance the reliability of bioprinted constructs, supporting clinical translation through standardized and reproducible quality assurance processes.

## 6 Integration of AI and 3D Bioprinting with Focus on TME Modeling

The integration of AI with 3D bioprinting for developing TME models represents a rapidly evolving interdisciplinary field, merging innovations from AI, 3D bioprinting, and oncology research. While AI and 3D bioprinting have been independently recognized as transformative technologies, their convergence in the specific context of cancer modeling remains at an early stage of development.

Advanced technologies such as MRI, CT imaging, high-throughput screening, automated screening techniques, and multiplex immunohistochemistry, when coupled with AI, play a crucial role in tumor bioprinting. These tools enable precise preprocessing, allowing for accurate simulation of the TME and the creation of more realistic 3D bioprinted tumor models. Such models provide a valuable platform for studying tumorigenesis, biophysical properties, and tumor-stroma interactions within a controlled and reproducible environment.

Despite this potential, the current body of research explicitly integrating AI-driven methodologies with 3D bioprinting for TME modeling remains extremely limited. Within the reviewed literature, only one original research article directly addressed this integration. In this pioneering study, Tang et al. utilized DLP bioprinting combined with ML algorithms to enhance the evaluation and understanding of glioma treatment responses (Figure 10).^199^ This work exemplifies the advantages of merging AI and bioprinting technologies, enabling not only the fabrication of more physiologically relevant glioma models but also the advanced analysis of treatment efficacy within complex TMEs.

**Figure 10:**
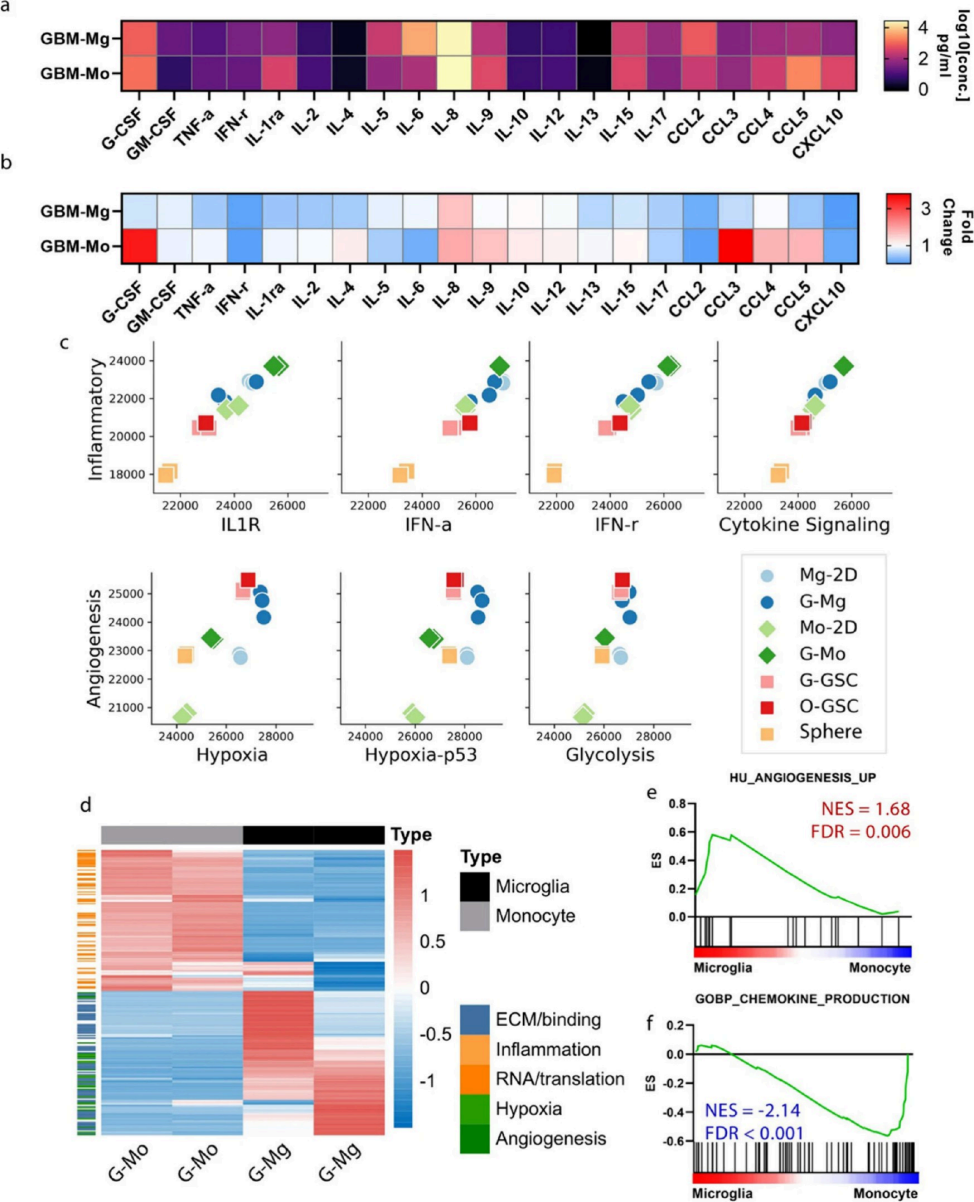
(A) Absolute cytokine abundance in GBM-Mg and GBM-Mo models. (B) Fold-change comparison of cytokine abundance in coculture supernatants vs supernatants from monocultures of the corresponding cell types. (C) GlioML feature analysis of cells isolated from bioprinted GBM-myeloid models and their traditionally cultured counterparts. (D) Heatmap representation of the top differentially expressed genes in G-Mg and G-Mo, with related pathways annotated on the side. (E) GSEA showing greater enrichment of the angiogenesis pathway in G-Mg. (F) GSEA demonstrating enhanced chemokine production pathway in G-Mo. Reproduced from ref 199. Available under a CC-BY 4.0 license. Copyright 2024 Tang et al.

Tang et al.’s study focused on the glioma microenvironment, a particularly challenging and clinically significant area in oncology. GBM and related gliomas are notorious for their aggressive behavior, resistance to therapy, and highly heterogeneous TME. These complexities make gliomas ideal candidates for the application of advanced bioprinting and AI strategies. By integrating ML, the study facilitated detailed predictions of tumor response to T cell-based and antiangiogenesis treatments and revealed a distinct immunosuppressive and angiogenic TME driven by myeloid infiltration. These insights demonstrate the potential of such models to inform precision oncology strategies and accelerate the identification of novel therapeutic targets.

However, the scarcity of studies explicitly combining AI with 3D bioprinting for TME modeling underscores a critical gap in the current research. There is a pressing need for further interdisciplinary efforts to expand this area, particularly across other tumor types beyond glioma. Future research should focus on adapting these integrative methodologies to cancers with similarly complex microenvironments such as pancreatic, colorectal, and breast cancers. Additionally, standardization of AI-based workflows, integration of multiomics data, and validation of in vitro findings in clinical contexts are essential to realize the full potential of these technologies.

In conclusion, glioma serves as a proof-of-concept for the integration of AI and 3D bioprinting in TME modeling. Its selection as the first and, so far, only example likely stems from the clinical urgency and the inherent complexity of the glioma microenvironment, which necessitates more sophisticated modeling strategies. This highlights both the opportunities and current limitations of the field, reinforcing the need for sustained research to translate these advances across a broader spectrum of oncological applications.

## 7 Summary, Perspectives, and Challenges

Colorectal cancer, oral cancer, breast cancer, and glioma remain significant global health challenges, occurring either sporadically or as a result of hereditary mutations. Personalized treatment strategies are increasingly implemented, especially in advanced or metastatic cases, to improve patient outcomes. A key conclusion of this review is that 3D bioprinting offers a promising pathway for constructing physiologically relevant tumor models that better replicate the complexity of the TME. Furthermore, the integration of AI into 3D bioprinting workflows enhances precision, efficiency, and reproducibility, thus advancing research on tumor biology, drug screenpaping, and personalized therapies.

Despite the progress made, the integration of AI with 3D bioprinting in cancer research remains at an early stage, with glioma currently serving as the most advanced proof-of-concept model. The main challenges ahead involve improving model standardization, expanding applications beyond glioma to other cancers, and validating these technologies in clinically relevant settings. Future perspectives point toward the development of more sophisticated, patient-specific models through AI-enhanced bioprinting with the potential to transform both preclinical research and personalized oncology.

### 7.1 Challenges and Limitations of 3D Printing in Cancer Research

Despite its significant potential, several limitations continue to hinder the widespread adoption of 3D-printed cancer models in both research and clinical practice:

#### 7.1.1 Regulatory Challenges

Currently, there is a lack of clear and unified regulatory frameworks for the use of 3D-printed biomaterials in oncology. While the FDA has conducted preliminary assessments of the technology and materials, clear legal frameworks for additive manufacturing in cancer research and treatment are still lacking.

#### 7.1.2 Limited Commercialization

Applications of 3D bioprinting in oncology remain largely confined to academic and research settings. The commercial-scale production of biofabricated materials tailored specifically to cancer therapies has yet to be realized.

#### 7.1.3 Manufacturing and Standardization Hurdles

The production processes for both simple and complex bioprinted constructs require further optimization, standardization, and certification. Additionally, the sector lacks specialized companies focused on manufacturing, distributing, and maintaining 3D bioprinting solutions for oncology.

#### 7.1.4 High Costs

The high financial costs associated with both 3D bioprinting and AI-enhanced modeling technologies currently limit their broader adoption in clinical practice.

#### 7.1.5 Lack of Scalability

Ensuring robust clinical validation and scalability of 3D-printed tumor models remains a key challenge in translating this technology into mainstream oncology.

#### 7.1.6 Incomplete Validation

Results generated from 3D-printed tumor models must be rigorously compared with traditional preclinical models, such as animal studies, to ensure accuracy, reproducibility, and reliability before clinical translation can be considered.

#### 7.1.7 Data-Related Challenges

A further limitation concerns the quality, availability, and standardization of data sets required to effectively integrate AI with 3D bioprinting in TME modeling. AI model performance and reproducibility depend strongly on well-annotated, standardized data; however, existing data sets are often small, fragmented, and inconsistently reported across studies. Data imbalance and heterogeneity may introduce bias during model training, limiting the predictive performance. Addressing these challenges will require the establishment of open-access, curated repositories and the adoption of community-wide standards for data acquisition, reporting, and sharing. Without such advances in data management, the reliable application of AI in bioprinted TME models will remain constrained.

### 7.2 Future Perspectives

Despite the current limitations outlined in Section 7.1, the future integration of 3D bioprinting and AI in CRC, oral cancer, BrCa, and glioma research remains highly promising. To overcome regulatory gaps, future efforts should prioritize the development of standardized protocols for bioink formulation, bioprinting processes, and the validation of 3D-printed constructs. Establishing these standards will facilitate the translation of laboratory advances into clinically relevant applications.

Technological innovations, particularly AI-driven optimization of printing parameters and bioink properties, offer potential solutions to current scalability and reproducibility issues. For example, ML models could support the standardization of manufacturing processes, ensuring the consistent production of complex, viable tissue constructs while minimizing cost and variability. AI could also play a central role in regulatory approval processes by providing validated, data-driven evidence of construct performance and reproducibility.

Further, AI-based predictive models will enhance the biological fidelity of printed TME, improving drug screening platforms and supporting personalized therapeutic strategies. Advances in digital twin technologies and in silico modeling will enable the design of individualized, patient-specific cancer models, bridging the gap between experimental oncology and clinical practice.

In the coming years, the convergence of AI and 3D bioprinting is expected to address current challenges by enhancing predictive accuracy, streamlining production workflows, and refining validation methodologies. These advancements will accelerate the clinical translation of 3D-printed TME models, improving diagnostic precision, treatment planning, and therapeutic outcomes across all four cancer types discussed in this review.

## Supplementary Material



## Notes

Boolean search expressions used for the literature search (January 2020–June 2025) (PDF)

## Abbreviations

2PP: Two-photon polymerization
3DP: Three-dimensional printing
ADMSCs: adipose-derived mesenchymal stem cells
AI: Artificial intelligence
BrCa: Breast cancer
CAFs: Cancer-associated fibroblasts
CNNs: Convolutional neural networks
Col1: Collagen type I
CRC: Colorectal cancer
CT: Computed tomography
DBB: Droplet-based bioprinting
DL: Deep learning
DLP: Digital light processing
EBB: Extrusion-based bioprinting
ECM: Extracellular matrix
EHS: Engelbreth–Holm–Swarm
GelMA: Methacrylated gelatin
GBM: Glioblastoma
HA: Hyaluronic acid
HCC: Hepatocellular carcinoma
HNC: Head and neck cancers
JBB: Jetting-based bioprinting
LAB: Laser-assisted bioprinting
LOX: Lysyl oxidase
ML: Machine learning
MMPs: Matrix metalloproteinases
MRI: Magnetic resonance imaging
MSCs: Mesenchymal stem cells
OSCC: Oral squamous cell carcinoma
SLA: Stereolithography
TECs: Tumor-associated endothelial cells
TME: Tumor microenvironment
VPB: Vat photopolymerization-based bioprinting
