# Supplementary material for: 3D Bioprinting and Artificial Intelligence for Tumor Microenvironment Modeling: A Scoping Review of Models, Methods, and Integration Pathways
Source: Mol Pharm. 2025 Sep 18;22(10):5801–23. doi: 10.1021/acs.molpharmaceut.5c01062 (PMC12505267; doi:10.1021/acs.molpharmaceut.5c01062)
Supplement: Supplementary file 1 [file mp5c01062_si_001.pdf]

3D Bioprinting and Artificial Intelligence for Tumor Microenvironment Modeling: A Scoping Review of Models, Methods and Integration Pathways

Urszula Piotrowska[1*], James Tsoi[2], Pradeep Singh[2], Avijit Banerjee[3], Marcin Sobczak[1]

[1] Department of Pharmaceutical Chemistry and Biomaterials, Medical University of Warsaw, 1 Banacha Str., 02-097 Warsaw, Poland;

[2] Division of Applied Oral Sciences and Community Dental Care, Faculty of Dentistry, The University of Hong Kong, Hong Kong SAR 999077, China;

[3] Centre of Oral Clinical Translational Sciences, Faculty of Dentistry, Oral & Craniofacial Sciences, King's College London, London WC2R 2LS, United Kingdom

***Corresponding author:**

Urszula Piotrowska
Department of Pharmaceutical Chemistry and Biomaterials
Medical University of Warsaw
1 Banacha Str., 02-097 Warsaw, Poland
urszula.piotrowska@wum.edu.pl
https://orcid.org/0000-0002-2418-9528

Table S1. Boolean search expressions used for the literature search (January 2020 – June 2025).

**Section**	**Search string (Boolean expression)**
1. 3D bioprinting of TME models for selected cancer types	3D bioprinting AND ("tumor microenvironment" OR TME) AND (colorectal cancer OR oral cancer OR breast cancer OR glioma)
2. AI applications in 3D bioprinting (general scope)	3D bioprinting AND ("artificial intelligence" OR "machine learning" OR "deep learning")
3. AI-assisted modeling of the tumor microenvironment	3D bioprinting AND ("tumor microenvironment" OR TME) AND ("artificial intelligence" OR "machine learning" OR "deep learning")
